# Contemporary Theory and Research

**Published:** 1893-05-13

**Authors:** 


### Contemporary Theory and Research.

### THE PARISH DOCTOR IN GREECE.

Dr. Kesner has an interesting article in the *Medical Magazine* pointing out that numerous tests and epigraphs exist to show that in every Greek city there was a public physician, whose duty it was to attend gratuitously both strangers and citizens, though practically only the poor claimed the benefit of his free ministrations. The inscriptions and bas reliefs from which this fact is ascertained are preserved in the British Museum. They consist of ten votive tablets, kept under glass in a small room near the mausoleum, with the sepulchral tablet of Jason and certain popular decrees throwing considerable light on the status of the medical profession in Greece.

### STARTLING OPERATION.

From Paris comes an account of an operation performed by a young surgeon, M. Ricard, on a woman of forty-five, who had suffered from a tumour in the frontal bone. A portion of the bone had to be removed in order to get rid of the tumour, and as the brain was laid bare in consequence, cerebral hernia ensued. Mr. Ricard had the bold idea of removing a piece of living bone from a dog's skull, and cutting it exactly to fit into the void in the woman's forehead. He took very careful antiseptic precautions—the grafted piece was knit with the frontal bone, into which it was inserted, and the patient made a good recovery. Dr. Polaillon has presented a report on the case to the Académie de Medicine, and speaks of the operation as the first successful one of the kind ever performed, and marking the recent progress in surgery and antiseptic science.

### TOBACCO AS A MICROBICIDE.

Dr. Tassinari has published in the *Italia Termale* the results of an investigation into the effects of tobacco smoke on microbes. He finds: (1) That the smoke of the Cavour, Virginia, and Tuscan cigars, and all black and chopped tobaccos possess a very pronounced bactericide power, especially against the bacillus of Asiatic cholera. (2) This microbicide action may in all probability be attributed to the products of nicotine. (3) In epidemics of cholera and typhus the use of tobacco may be rather useful than hurtful. (4) Tobacco smoke merits special consideration on the hygiene of the mouth as a prophylactic means of combating microbian affections of the buccal cavity.

### DOG-SKIN GLOVES.

Dr. Puy le Blanc has contributed to the *Journal de Médicine* a report of a case of eczema of the hands, provoked entirely by the use of dog-skin gloves, coloured red to imitate Russian leather. By means of an analysis he discovered the substance used in dyeing the skins to be aurantia, and further investigations showed that the workmen employed in the dye works had been attacked by similar affections of the face and hands. Aurantia is obtained by the reaction of nitric acid on diphenylamine; it gives a deep orange tint, and to obtain red a minute portion of fuchsine is added. Its irritatant action is uncertain, some persons being entirely unaffected by it. In many factories the use of this dye has been discontinued, but while there is any chance of encountering such unpleasant effect, gloves of that particular reddish hue will be regarded with suspicion.

